# Development of a 1 × 512 Ring Transducer Array-Based 3D Ultrasound Imaging System for Accurate Breast Lesion Detection: Phantom and Preliminary Clinical Feasibility Study

**DOI:** 10.3390/mi17020223

**Published:** 2026-02-08

**Authors:** Zhaodi Hou, Fei Wu, Dan Gao, Renxin Wang, Guojun Zhang, Changde He, Jiangong Cui, Wendong Zhang, Yuhua Yang, Licheng Jia

**Affiliations:** State Key Laboratory of Extreme Environment Optoelectronic Dynamic Measurement Technology and Instrument, North University of China, Taiyuan 030051, China

**Keywords:** high resolution, three-dimensional ultrasound imaging system, 1 × 512 ring transducer array, optimal sound speed estimation, preliminary clinical feasibility

## Abstract

The work presents an algorithm for early detection of breast microlesions using a high resolution three-dimensional ultrasound imaging system. The system employs a 1 × 512 ring transducer array and a triaxial displacement platform with an accuracy of 0.1 mm, achieving high-density acquisition of three-dimensional volumetric data through fixed-step scanning. To improve imaging quality, an adaptive beamforming algorithm incorporating optimal sound speed estimation is proposed, effectively compensating for phase distortion caused by sound speed heterogeneity within tissues and improving spatial coherence and imaging resolution. The three-dimensional volumetric data is visualized using volume rendering to achieve high-fidelity three-dimensional ultrasound image reconstruction. The in vitro experimental results demonstrate that the proposed algorithm improves the system’s spatial resolution to 0.5 mm, with a linear measurement accuracy of 2.1%. A preliminary clinical feasibility case study comparing breast image reconstruction with MRI imaging results shows a Dice similarity coefficient of 0.87 for the lesion region, high anatomical structure reconstruction accuracy, and good spatial consistency. These results demonstrate preliminary clinical feasibility for early detection of breast microlesions.

## 1. Introduction

Breast cancer remains the most prevalent malignancy among women globally and continues to represent a significant public health challenge [1,2]. Early and precise detection of lesions smaller than 5 mm is critical to improving patient prognosis and survival rates [3]. Currently, commonly used imaging modalities in clinical settings include X-ray mammography, handheld ultrasound, and magnetic resonance imaging (MRI) [4]. While these techniques play crucial roles in clinical diagnosis, each has inherent drawbacks that restrict their widespread and repeatable application. Specifically, X-ray mammography involves exposure to ionizing radiation, limiting its suitability for frequent monitoring; handheld ultrasound results are highly operator-dependent, leading to variability in interpretation; and although MRI provides excellent soft-tissue contrast, it is associated with high costs, limited accessibility, and patient contraindications (e.g., claustrophobia), making it unsuitable for broad population screening [5,6,7].

In order to overcome the limitations of existing methods, an ultrasound computed tomography imaging method that offers the advantages of being non-invasive and convenient, using non-ionizing radiation, and being relatively inexpensive was developed [8,9]. Ultrasound reflection tomography can provide specific information about a particular facet of the breast, but it cannot describe the spatial localization and orientation of the internal structure of the breast tissue, whereas the reconstruction of 3D imaging can make up for the shortcomings of tomography, enabling the analysis of the characteristic volume and shape of interest [10]. Therefore, 3D ultrasound data reconstruction techniques have been intensively investigated [11,12], and 3D imaging methodology techniques have been used to inform medical imaging diagnosis. 3D ultrasound imaging aims to overcome these challenges by providing volume data sets and image resolution. However, existing 3D ultrasound imaging systems are limited by mechanical localization errors, poor image reconstruction algorithms, and anisotropic voxel sizes, which result in ultrasound imaging systems with low spatial resolution [13]. Specifically, the ultrasound image reconstruction process produces phase aberrations due to the heterogeneity of the lesion tissue, and thus, discriminating between a target with a small size and two targets with a small separation distance remains a challenge [14,15].

In order to improve the resolution of the ultrasound imaging system, a high-resolution three-dimensional ultrasound imaging system for early breast cancer screening was constructed in this paper. The system adopts a two-layer detection bed design, with the upper bed plate opening an oval-shaped opening corresponding to the thoracic cavity for the breast to naturally drape when the subject is lying down. The lower layer integrates a three-axis displacement stage system to drive the ring transducer array and water cylinder to position to the breast projection area, and vertically lifts and lowers the breast tissue to realize the immersion of the water-coupled medium to ensure the stable transmission of acoustic waves and layer-by-layer data acquisition. A multi-aperture beam synthesis algorithm with optimal sound speed estimation was used for image reconstruction of the data, while body mapping was used to visualize the three-dimensional body data, and the reconstruction results showed improved image resolution.

This study employed in vitro experiments to validate the performance of the constructed 3D ultrasound imaging system, and the results demonstrated significant improvements in both spatial resolution and target size reconstruction accuracy. Furthermore, the system was evaluated in a preliminary clinical feasibility case study, where its imaging outcomes were compared with those obtained from enhanced magnetic resonance imaging (MRI).

## 2. Theory

### 2.1. Delay-Summing Beamforming Algorithms in Ultrasound Reflectance Imaging

The basic principle of ultrasound imaging is that ultrasound waves are sent from the transmitting array to the target area and then reflected back and captured by the receiving array, and the relationship between the acoustic signal and the acoustic impedance distribution [16] is deduced by processing the received raw echo signals [17] to reconstruct the image. The delay generated by the ultrasonic waves in the propagation path during data acquisition can have an impact on the aberration [18,19]. Therefore, the delayed summation beamforming (DAS) algorithm is an important step in the ultrasound imaging process, which can realize the summing of coherent signals of the array elements in order to improve the target resolution. The basic principle is to compensate for the time of flight of the echoes received by different array elements and then align the signals to reduce the interference caused by the time difference.

The 3D ultrasound imaging system uses a ring transducer array of uniformly distributed array elements to detect the target region from multiple directions, and the propagation delay between the transmitting and receiving array elements to the imaging point is calculated by defining the center of the ring transducer array as the origin of the Cartesian coordinate system. Assuming that a point P(*x*,*y*,*z*) on the imaging grid is our point of interest, let the coordinates of the transmitting array element Tx and the receiving array element Rx be ($x_{Tx}$,$y_{Tx}$) and ($x_{Rx}$,$y_{Rx}$), respectively. Then, the distances from the imaging point P to the transmitting array element Tx and receiving array element Rx are:

(1) $$d_{Tx}\left(P\right)=\sqrt{{\left(x-x_{Tx}\right)}^{2}+{\left(y-y_{Tx}\right)}^{2}+z^{2}}$$

(2) $$d_{Rx}\left(P\right)=\sqrt{{\left(x-x_{Rx}\right)}^{2}+{\left(y-y_{Rx}\right)}^{2}+z^{2}}$$

where *z* is the depth coordinate of the imaging point. With these two distances, the signal propagation time $t_{Tx}\left(P\right)$ from the transmitting array element Tx to the imaging point *P* and the signal propagation time $t_{Rx}\left(P\right)$ from the receiving array element Rx to the imaging point *P* can be calculated.

The transmitted and received propagation times can be expressed as:

(3) $$t_{Tx}\left(P\right)=\frac{d_{Tx}\left(P\right)}{c}$$

(4) $$t_{Rx}\left(P\right)=\frac{d_{Rx}\left(P\right)}{c}$$

where the speed of sound is *c*, while the sampling point $t_{idx}$ corresponding to the propagation delay is:

(5) $$t_{\mathrm{idx}}=\left(\frac{d_{Tx}\left(P\right)+d_{Rx}\left(P\right)}{c}\right)\times f_{s}$$

where $f_{s}$ is the sampling frequency.

In the beam forming process, it is necessary to weight and superimpose the signals of each receiving array element at its sampling point. Assuming that the received signal of the *n* transmitting array element at the receiving array element *m* is $r_{n,m}\left(t\right)$, then for the imaging point P(*x*,*y*,*z*), the signal after weighted superposition can be expressed as:

(6) $$focData\left(x,y\right)=\sum_{m-1}^{N}\sum_{n-1}^{N}w_{m,n}\left(x,y\right)r_{n,m}\left(t_{m,n}\left(x,y\right)\right)$$

where *N* is the total number of elements in the transducer array; $w_{m,n}\left(x,y\right)$ is the dynamic weighting coefficient of the imaging point (*x*,*y*) corresponding to the nth transmitting element and the mth receiving element; $r_{n,m}\left(t\right)$ is the received signal of the nth transmitting element at the mth receiving element; and $t_{m,n}\left(x,y\right)$ is the delay time of the imaging point (*x*,*y*) corresponding to the nth transmitting element and the mth receiving element.

### 2.2. Optimal Sound Speed Finding Based on Coherence Factor

In ultrasound imaging, the exact sound speed setting has a critical impact on image clarity and target localization accuracy. Due to the heterogeneity of biological tissues, the actual sound propagation speed often deviates from the ideal assumed value (e.g., 1540 m/s). This mismatch can result in a range of imaging issues, including focal point displacement, image blurring, and increased artifact formation, ultimately affecting image quality and diagnostic accuracy. The coherence factor is a representative method for evaluating the coherence of received signals [20]. Accordingly, the optimal sound speed within the region of interest can be estimated by maximizing the coherence coefficient, which serves as a criterion for phase aberration correction [21,22].

In this study, an optimal sound speed estimation method based on L1 Coherence Factor (L1CF) is used to determine the optimal propagation sound speed by evaluating the coherence of the reconstructed image at different candidate sound speeds. L1CF is a measure of signal coherence during beam synthesis, which is widely used in the adaptive beam forming and image quality assessment fields. It is defined as follows:

(7) $$L1CF\left(r\right)=\frac{{\left|\sum_{i=1}^{N}s_{i}\left(r\right)\right|}^{2}}{\sum_{i=1}^{N}{\left|s_{i}\left(r\right)\right|}^{2}+\epsilon }$$

where $r\in R^{3}$ denotes the coordinates of a point in the imaging region, $S_{i}\left(r\right)$ denotes the delay-compensated RF signal of the *i* channel at the point, *N* is the number of receiver channels participating in the synthesis, and ϵ is a very small positive number preventing the denominator from being zero. The specific explanation is that if the signals of each channel are well aligned at the target point and the propagation time compensation is accurate, the amplitude superposition will be close to the sum of the amplitudes, and therefore the L1CF will be close to 1. On the contrary, if the sound speed value is chosen with a large error, the phase deviation between the channels will cause the interference, resulting in a decrease of the L1CF.

### 2.3. Body-Mapped 3D Ultrasound Imaging Reconstruction

3D ultrasound imaging reconstruction requires the extraction of image information from a series of ultrasound reflection tomography images to obtain 3D body data for the reconstruction model [23]. Body mapping is a fundamental technique for visualizing 3D ultrasound datasets and enables the synthesis of 2D projected images from body data, ensuring detailed information and tissue continuity in 3D reconstruction results [24]. Body mapping mainly uses the Ray Casting algorithm to simulate the process of light penetration into the body data [25], and the 3D reconstruction results are based on the density difference between different tissues in response to different levels of brightness and darkness of the mapping to determine the normal tissues and lesions [26].

The 3D ultrasound imaging reconstruction is done by obtaining the pixel value (i,j) at each coordinate position of each imaging result for each layer, which is represented in 3D coordinates as:

(8) $$X_{ijk}=IPP_{k}+i\cdot \Delta_{r}\cdot r+j\cdot \Delta_{c}\cdot c$$

where *r* and *c* are the unit vectors in the row and column directions of the image, Δ*r* and Δ*c* are the pixel spacing in the row and column directions and *k* is the slice index. The acquired data are stacked to form the body data by the calculation of the above equation. In order to ensure the resolution of the three-bit ultrasound reconstruction results, a linear interpolation method is used to estimate the value of a certain position in the body data with the following mathematical expression:

(9) $$V\left(x,y,z\right)\approx \sum_{a=0}^{1}\sum_{b=0}^{1}\sum_{c=0}^{1}w_{abc}\cdot V\left(i+a,j+b,k+c\right)$$

using the weights:

(10) $$w_{abc}=\left(1-|x-\left(i+a\right)|\right)\cdot \left(1-|y-\left(j+b\right)|\right)\cdot \left(1-|z-\left(k+c\right)|\right)$$

where V(i+a,j+b,k+c) is the voxel value of the neighboring 8 voxels.

Finally, each ray passing through the voxel data is weighted fused to the voxel with the mathematical expression:

(11) $$I=\int_{t_{0}}^{t_{1}}\alpha \left(r(t)\right)\cdot V\left(r(t)\right)\cdot e^{-\int_{t_{0}}^{t}\alpha \left(r(s)\right)ds}dt$$

where is the transparency mapping function and r(t) and r(s) are the spatial paths of the rays in the body data.

## 3. Methods

### 3.1. 1 × 512 Ring Transducer Array 3D Ultrasound Imaging System

**Figure 1:**
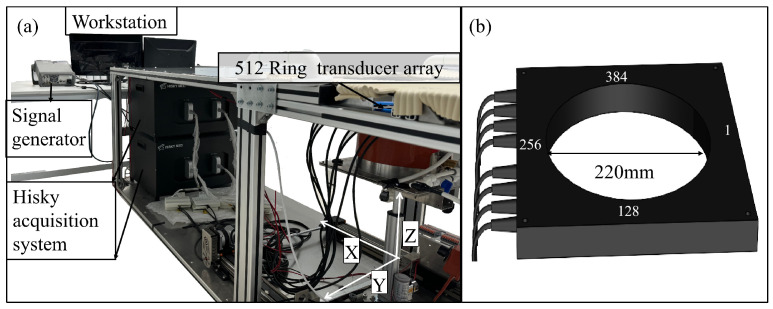
Schematic diagram of the 1 × 512 ring transducer array-based 3D ultrasound imaging system: (**a**) Overall system structure, including the computer workstation, signal generator, Hisky acquisition system, 1 × 512 ring transducer array, and three-axis stage; (**b**) A 1 × 512 ring transducer array with a diameter of 220 mm.

To advance beyond the capabilities of the previously developed 256-channel system [14] to the 512-channel system, we developed a 512-channel three-dimensional ultrasound imaging system. The main components and connection method of this system are shown in Figure 1a. This ultrasound imaging system consists of a 1 × 512 ring transducer array, two data acquisition systems (Wuxi Hisky, Wuxi, China), a computer workstation, and a three-axis stage. The ring transducer array is composed of 512 single elements, as shown in Figure 1b. The 512 ring transducer array uses the same center frequency (3 MHz), bandwidth (−6 dB) ≥ 60%, homogeneity in sensitivity (±3 dB), and elevation (10 mm) as the 256 ring transducer array previously studied by our research group. The difference lies in the elementary pitch (2.454 mm), the element interspace (0.2 mm), and the array radius (100 mm) of the 256 ring transducer array, while the 512 ring transducer array has a lower elementary pitch (1.35 mm) and element interspace (0.1 mm), and a higher array radius (110 mm).

The ring transducer reduces incoherent signals caused by crosstalk by adding a shielding layer between two array elements and selecting a suitable matching layer. To improve signal fidelity and reduce system complexity, we adopted a parallel acquisition architecture, using two identical Hisky acquisition systems operating in coordination. Multi-channel parallel processing enhances data processing capabilities. The data acquisition process uses a square wave bipolar pulse waveform with 5 complete cycles and an AC voltage of 90Vpp for excitation. A signal generator simultaneously triggers both acquisition systems, achieving 512-channel transmit/receive control in a one-to-all mode. The acquisition time per frame is approximately 0.063 s, a reduction of about 0.077 s compared to the previously developed 256-channel acquisition time.

A high-performance workstation with a GPU is used for offline data processing to further shorten reconstruction time. The three-axis stage has a positioning accuracy of 0.1 mm, precisely positioning the subject along the X and Y axes to ensure accurate alignment with the transducer array center. Simultaneously, the Z-axis moves the subject from bottom to top at uniform intervals and a constant speed, achieving vertical volumetric ultrasound reflection tomography data acquisition. This process provides high-quality input data for subsequent 3D image reconstruction.

### 3.2. Introduction to the Experiment

**Figure 2:**
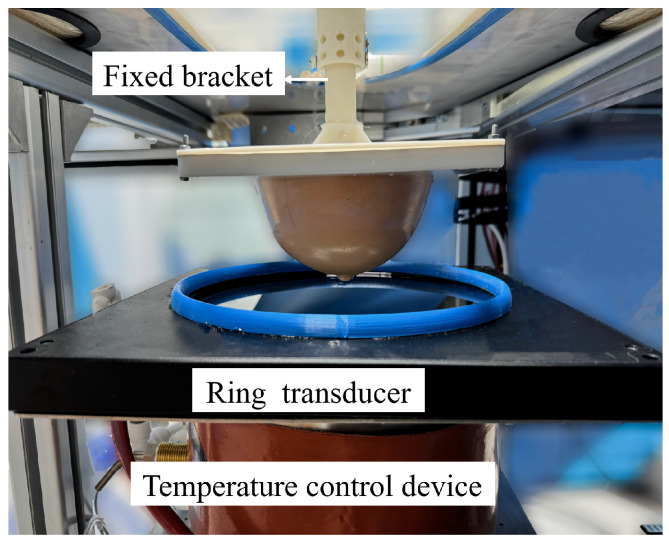
Data acquisition device.

To verify the resolution of this 3D ultrasound imaging system, we used a customized KS107BHF resolution ultrasound model and a KS105-HFR tissue ultrasound simulation phantom from the Institute of Acoustics, Chinese Academy of Sciences, for analysis and verification. The ring transducer array was connected to a water tank via a fixed component, and a temperature control device was installed outside the tank to maintain a constant internal temperature, ensuring that the acoustic coupling between the ring transducer array and the tested phantom was completely submerged in water during the experiment. The experimental setup is shown in Figure 2. A sampling frequency of 40 MHz was set to increase the number of sampling points and improve signal fidelity.

**Figure 3:**
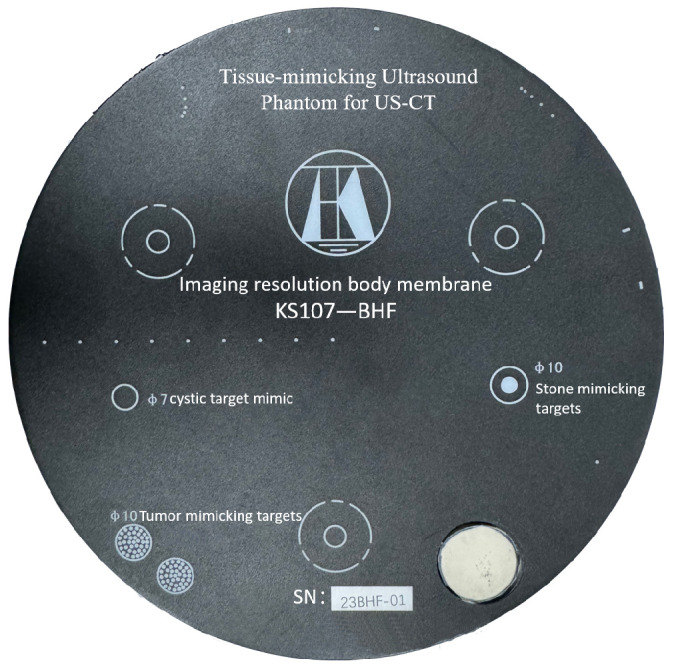
Model KS107BHF resolution ultrasound die body.

The Model KS107BHF Imaging Resolution Phantom presents a cylindrical structure in appearance, which has a plurality of target groups composed of simulated tumor-like lesions, cystic-like lesions, stone-like lesions, axial and lateral resolution target line clusters, and a single target line. The axial resolution target group consists of five parallel target lines, with inter-line spacings set at 4 mm, 3 mm, 2 mm, and 1 mm, respectively. Similarly, the lateral resolution target group comprises five target lines with spacings of 3 mm, 2 mm, 1 mm, and 0.5 mm. The diameter of each individual target line in both groups is 0.3 mm. In addition, the phantom includes two tumor-mimicking lesions, each with a diameter of 10 mm, two cyst-mimicking lesions with diameters of 7 mm, and one stone-like lesion with a diameter of 10 mm. In addition, the interior contains three support columns with acoustic absorption properties arranged in a triangular shape with a diameter of about 20 mm. The imaging resolution phantom of the KS107BHF model is shown in Figure 3, and the detailed specifications and the speed of sound are described in Table 1. The 3D ultrasound imaging system was used to perform 3D reconstruction of the KS107BHF-type imaging resolution phantom, and the triaxial displacement elevation platform was used to acquire 30 layers of data at 1 mm intervals to obtain 3D body data for reconstruction.

**Figure 4:**
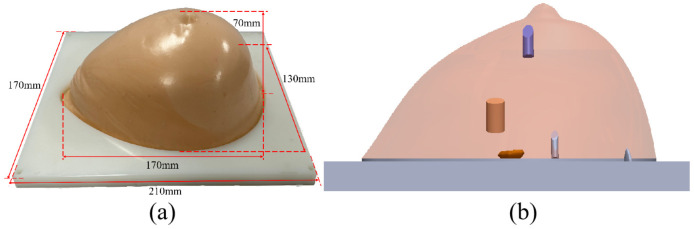
KS105-HFR phantom and lesion distribution overview: (**a**) Model KS105-HFR breast tissue-mimicking phantom; (**b**) Physical phantom internal structure distribution design diagram.

The appearance of the KS105-HFR breast tissue-mimicking phantom is in the shape of a mammary gland as shown in Figure 4a, and its dimensions are 170 mm (L) × 130 mm (W) × 70 mm (H), and the size of the base plate of the phantom is 210 mm (L) × 170 mm (W). Figure 4b shows the internal structure design of the physical phantom. The model contains mimick cystic foci, mimick tumorous foci, and mimicking calcified foci, which are columnar or irregular in shape. The dimensions and sound speed data of different lesions are shown in Table 2.

In order to verify the reconstruction effect of this 3D ultrasound imaging system on irregularly shaped subjects, 3D reconstruction of the KS105-HFR breast tissue-mimicking phantom with a mammary gland appearance was performed. A three-axis displacement stage was used to vertically elevate the ring transducer array from the nipple to the thoracic wall portion at 1-mm intervals, and a total of 30 layers of data were acquired to reconstruct the KS105-HFR tissue-mimicking ultrasound phantom in three-dimensional modeling.

**Figure 5:**
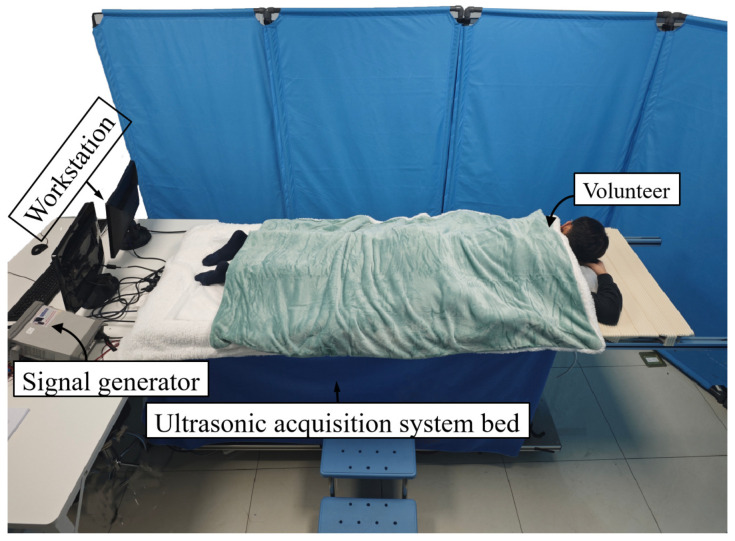
Clinical trials.

In addition, the 3D ultrasound imaging system was subjected to a preliminary clinical feasibility case study on recruited volunteers at the General Hospital of the Chinese People’s Liberation Army in Beijing, China. Before the preliminary clinical feasibility case study, an ethical application was first submitted to the relevant ethics committee, which included the study background, purpose, methodology, potential risks, protection measures for the volunteers, and an informed consent form, to ensure that the study design complied with the ethical requirements, and was ethically approved. In order to verify the preliminary clinical feasibility case study of the three-dimensional ultrasound imaging system, volunteers were recruited at the General Hospital of the Chinese People’s Liberation Army in Beijing, China, and the data collection method of the volunteers’ preliminary clinical feasibility case study is shown in Figure 5. The unilateral breast data collected from one of the volunteers was acquired, and the ring transducer array was ascended from the tumor portion to the chest wall at 2-mm intervals using a three-axis displacement stage, and a total of 23 layers of data were acquired to reconstruct the 3D model of the breast tumor portion.

### 3.3. Assessment of Indicators

The imaging quality of the system was evaluated using the spatial resolution of the image [27], and the target region relative sound intensity was extracted based on the difference in relative sound intensities of the target and the background region for data processing. The image spatial resolution is obtained by calculating the FWHM in the corresponding direction of the target, and then half of the peak relative sound intensity is selected from the point spread function curve to calculate the size of the target; the spatial resolution is determined according to the Rayleigh criterion, and the mathematical expression is as follows [28]:

(12) $$FWHM=2\sigma \sqrt{2\ln2}$$

where σ denotes the standard deviation of the signal.

To quantify the discrimination capability of different imaging methods for the target region, we adopted contrast-related metrics for evaluation. Among these metrics, the Contrast Ratio (CR) is utilized to quantify the mean intensity difference between the target region and the background region. It is defined as the ratio of the mean intensity of the target region to that of the background region, thereby reflecting the saliency of the target. Its formula is given as follows [29]:

(13) $$C=\frac{\mu_{i}}{\mu_{o}}$$

(14) $$\mu_{i}=E\{|s_{i}{|}^{2}\}$$

(15) $$\mu_{o}=E\{|s_{o}{|}^{2}\}$$

$\mu_{i}$ denotes the mean signal intensity of the target region, $\mu_{o}$ denotes the mean signal intensity of the background region, and *s* represents the signal value.

The generalized Contrast-to-Noise Ratio (gCNR) is employed to comprehensively account for the overlapping intensity distributions between the target and the background. Computed based on the cumulative distribution functions of the intensity histograms for the target and background regions, this metric enables a more accurate evaluation of target separability. The gCNR ranges from 0 to 1, and a value closer to 1 indicates stronger target detectability. Its formula is given as follows [29]:

(16) gCNR=1−OVL.

(17) $$OVL=\int \min(p_{i}\left(x\right),p_{o}\left(x\right))dx$$

OVL denotes the overlapping area of the probability density functions of the two regions. $p_{o}$ and $p_{i}$ represent the prior probabilities assigned according to the size of the region of interest.

The Dice coefficient was used to assess the degree of similarity of the lesions in the ultrasonography results and the enhanced MRI results, and a higher Dice coefficient indicates a greater similarity between the two samples [30], with the following mathematical expression:

(18) $$\mathrm{Dice}\left(A,B\right)=\frac{2|(A\cap B)|}{\left|A|+|B\right|}$$

A and B denote the set of pixels in the image, respectively.

## 4. Results

### 4.1. Resolving Power Verification

**Figure 6:**
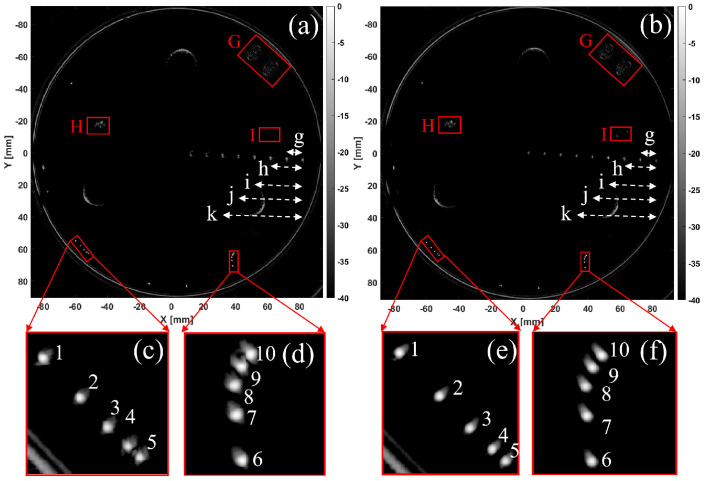
Results of KS107BHF resolution phantom reconstruction under different sound speeds: (**a**) Reconstruction of resolution phantom at 1540 m/s; (**b**) Reconstruction of resolution phantom at 1513 m/s; (**c**) Axial resolution target group results at 1540 m/s; (**d**) Lateral resolution target group results at 1540 m/s; (**e**) Axial resolution target group results at 1513 m/s; (**f**) Lateral resolution target group results at 1513 m/s.

In this section, this 3D ultrasound imaging system was used to acquire data and images and analyze the imaging resolution phantom of type KS107BHF. Based on the optimal sound speed estimation, the sound speed was determined to be 1513 m/s. Figure 6a shows the reconstruction results of the KS107BHF-type imaging resolution phantom using the conventional assumed sound speed of 1540 m/s, while Figure 6b presents the reconstruction results using the optimized sound speed of 1513 m/s. In the axial resolution targets (Figure 6c,e), targets 4 and 5 in Figure 6c are barely distinguishable. In Figure 6e, targets 4 and 5 are clearly separated. For the lateral resolution targets (Figure 6d,f), targets 9 and 10 are not distinguishable in Figure 6d, but they are clearly distinguishable in Figure 6f. Analysis of Figure 6b shows that the imaging results of the stone lesion (I) and cystic lesion (H) exhibit incomplete arcs, which is attributed to the acoustic absorption effects of the triangularly arranged support columns. The location and size of the two tumor-mimicking lesions (G), each with a diameter of 10 mm, closely match their actual dimensions.

Overall, the target reconstruction results at a sound speed of 1513 m/s show significantly better size and resolution than those at a sound speed of 1540 m/s. Therefore, using the optimal sound speed to locate the number, location, and morphology of targets within the KS107BHF phantom shows better consistency with the preset parameters.

To analyze the resolution of the target and background regions in reconstructed images under different sound velocities, we selected two sets of targets with intervals of 1 mm and 0.5 mm for CR and gCNR analysis. The target regions were targets 4, 5, 9, and 10, and the background region was selected from the area near the targets. The analysis results are shown in Table 3. The results show that the CR and gCNR of the image reconstructed using the optimal sound velocity estimation algorithm are improved compared to the image reconstructed using the assumed sound velocity, verifying the effectiveness of the optimal sound velocity estimation.

The CR and gCNR of lesions reconstructed at different sound velocities were analyzed. The target region was the lesion edge contour, and the background region was selected from the area near the lesion for analysis. The CR value under the assumed sound velocity was 8.33, and the gCNR value was 0.82; the CR value under the optimal sound velocity estimation was 15.83, and the gCNR value was 0.88. The results show that the CR and gCNR of the image reconstructed by the optimal sound velocity estimation algorithm are improved compared with those reconstructed by the assumed sound velocity, indicating that the target in the image reconstructed by the optimal sound velocity estimation algorithm is easier to identify.

**Figure 7:**
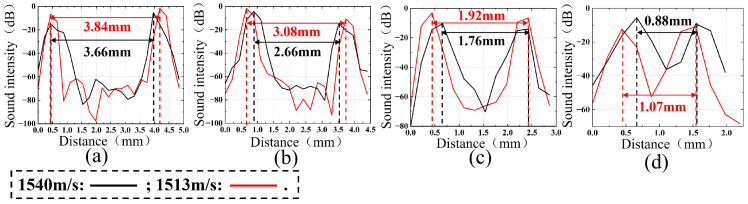
Results of target group spacing at axial resolution with sound speeds of 1540 m/s and 1513 m/s: (**a**) 1–2 target group spacing; (**b**) 2–3 target group spacing; (**c**) 3–4 target group spacing; (**d**) 4–5 target group spacing.

**Figure 8:**
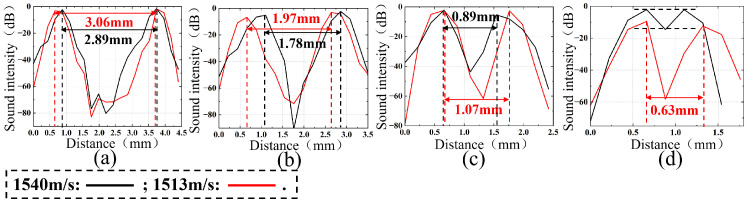
Results of target group spacing at lateral resolution with sound speeds of 1540 m/s and 1513 m/s: (**a**) 6–7 target spacing; (**b**) 7–8 target spacing; (**c**) 8–9 target spacing; (**d**) 9–10 target spacing.

Based on the distances between adjacent targets, the relative acoustic intensity information between adjacent targets was extracted separately for the axial resolution group (with distances of 4 mm, 3 mm, 2 mm, and 1 mm, as shown in Figure 6c,e) and the lateral resolution group (with distances of 3 mm, 2 mm, 1 mm, and 0.5 mm, as shown in Figure 6d,f). The analysis results are presented in Figure 7 and Figure 8, where the black line represents the distances at a sound speed of 1540 m/s, and the red line represents the distances obtained using the optimal estimated sound speed of 1513 m/s.

According to the Rayleigh criterion, the analysis results of the axial resolution targets are shown in Figure 7a–d. It can be seen that at a sound speed of 1540 m/s, the distance error between any two targets is greater than the distance error at the optimal estimated sound speed of 1513 m/s. When the distance decreases to 1 mm, the reconstruction result at a sound speed of 1540 m/s just resolves the target, while the reconstruction result at the optimal estimated sound speed of 1513 m/s achieves complete resolution.

According to the Rayleigh criterion, the analysis results for lateral resolution targets are shown in Figure 8a–d. Specifically, Figure 8a–c show the results when the distance is greater than or equal to 1 mm, at which point the two targets are completely distinguishable. Figure 8d shows the results for two targets at a distance of 0.5 mm. At a sound speed of 1540 m/s, the relative sound intensity difference between the two targets is very small, making them indistinguishable. In contrast, when using the optimal estimated sound speed of 1513 m/s, the two targets at a distance of 0.5 mm are completely distinguishable.

Table 4 presents the calibration spacing between the two targets and the reconstructed spacing and measurement error between the two targets at different sound speeds in a single experiment. The results in the table indicate that when the spacing between the two targets is greater than 1 mm, the spacing errors of the two targets reconstructed using the optimal estimated sound speed of 1513 m/s are all within 10%, which are significantly smaller than those reconstructed at 1540 m/s. When the spacing is 0.5 mm, the targets cannot be resolved at a sound speed of 1540 m/s, whereas they are distinguishable when the optimal estimated sound speed of 1513 m/s is adopted, with a corresponding error of 0.13 mm. The results show that using the optimal estimated sound speed of 1513 m/s can achieve a spatial resolution of 0.5 mm for the 3D imaging system.

**Figure 9:**
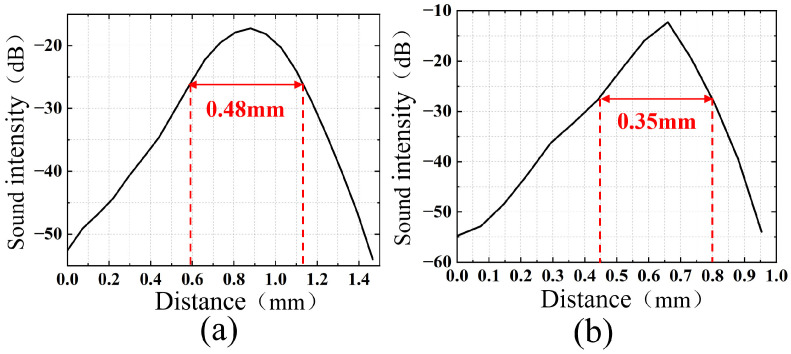
Quantization results for a 0.3 mm target. (**a**) Quantization result at a sound speed of 1540 m/s (**b**) Quantization result at a sound speed of 1513 m/s.

Based on the Rayleigh criterion, analysis and calculation were conducted on a single target line of 0.3 mm. The FWHM quantitative results are presented in Figure 9. Specifically, Figure 9a shows the reconstruction using a sound speed of 1540 m/s, with a reconstructed size of 0.48 mm and a reconstruction error of 0.18 mm. Figure 9b displays the reconstruction adopting the optimal estimated sound speed of 1513 m/s, where the reconstructed size is 0.35 mm and the reconstruction error is 0.05 mm. The results show that using the optimal estimated sound speed can reduce the error in the target reconstruction size from 0.18 mm to 0.05 mm.

Linear measurement accuracy is defined as the accuracy of performing linear distance measurements within an image slice, that is, distance from one point to another within an image [31]. Figure 6a,b show a set of imaging results for a resolution volumetric membrane under assumed and estimated sound velocities, respectively. g = 10 mm, h = 20 mm, i = 30 mm, j = 40 mm, and k = 50 mm represent five intervals for the resolution volumetric membrane. Table 5 shows the linear measurement accuracy analysis of the intervals reconstructed under two different sound velocity conditions. The results indicate that the average coefficient of variation and average percentage difference of the target intervals reconstructed using the optimal sound velocity estimation algorithm are lower than those reconstructed using the assumed sound velocity, demonstrating that the optimal sound velocity estimation algorithm improves measurement accuracy.

**Figure 10:**
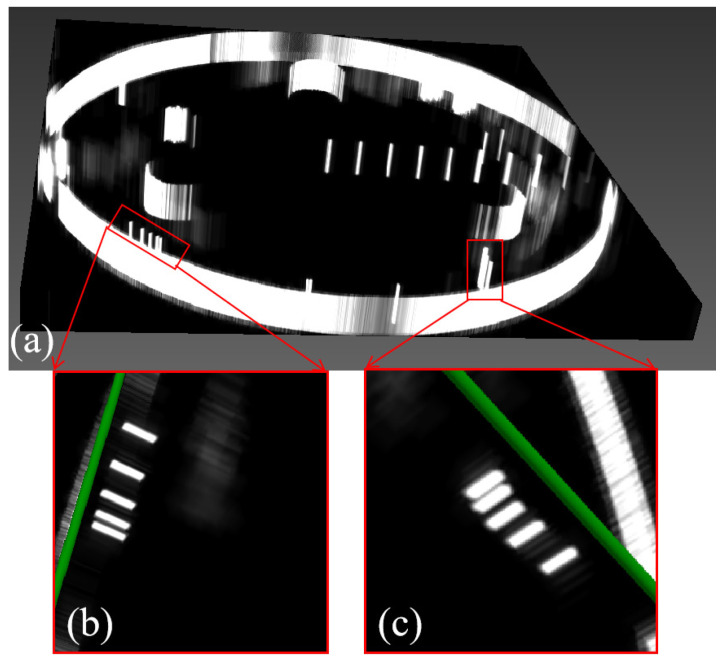
3D reconstruction and sagittal projections of resolution target groups. (**a**) 3D reconstruction results of the resolution voxel. (**b**) sagittal projection results of the axial resolution target group. (**c**) sagittal projection results of the lateral resolution target group.

The 3D reconstruction results are shown in Figure 10a. The axial resolution target group is projected in the sagittal plane as shown in Figure 10b, the lateral resolution target group is projected in the sagittal plane as shown in Figure 10c, and from the analysis of the 3D reconstruction results, the 3D ultrasound imaging system can discriminate target line intervals as small as 0.5 mm, and it can reconstruct a single target line of 0.3 mm. Using the constructed 3D ultrasound imaging system for tomographic imaging and 3D reconstruction analysis of the KS107BHF-type imaging resolution phantom, it can be concluded that the spatial resolution of this 3D ultrasound imaging system is 0.5 mm.

Compared with the target reconstructed by the three-dimensional reconstruction technique of breast multi-view ultrasound imaging based on linear array cylindrical motion proposed by Liu et al. [13] and the three-dimensional ultrasound imaging system based on a 1 × 256 ring transducer array developed by Zhang et al. [14], this system can achieve a smaller spatial resolution. Compared with the QT [31,32] and Softvue [33] ultrasound imaging systems, this system uses a relatively small number of ultrasound transducers to achieve sub-millimeter spatial resolution. Therefore, this system achieves a relative balance between imaging resolution and system complexity. Table 6 shows a comparison of the specific parameters of the core performance indicators in previous studies and this paper.

### 4.2. Mimicking Breast Somatic Phantom Imaging

**Figure 11:**
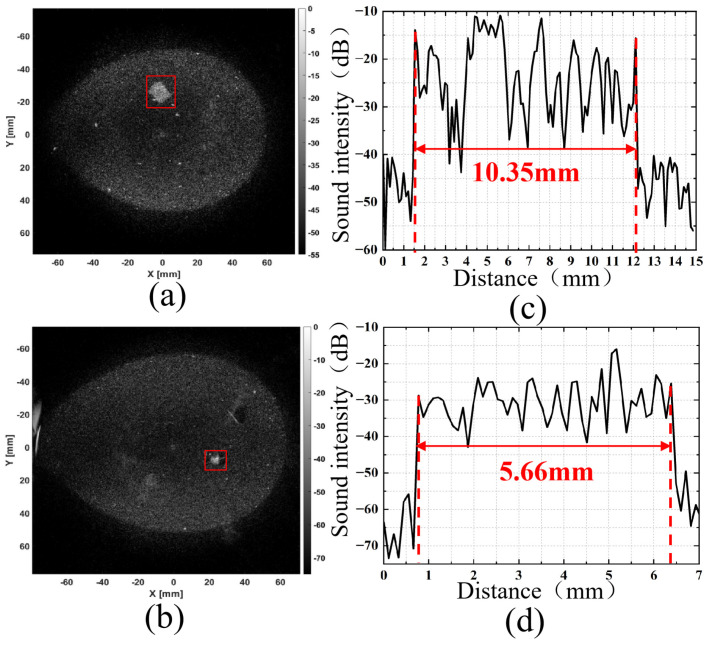
Imaging reconstruction and quantitative evaluation of 10 mm and 6 mm targets: (**a**) Reconstruction results at 10 mm target position; (**b**) Reconstruction results at 6 mm target position; (**c**) 10 mm target quantization results; (**d**) 6 mm target quantization results.

**Figure 12:**
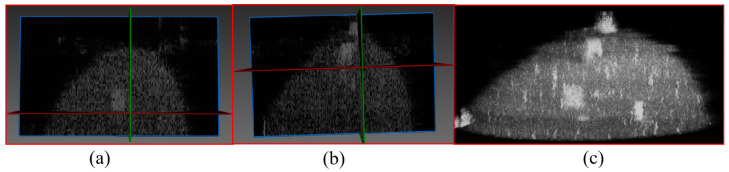
Coronal projections and 3D reconstruction of targets and breast tissue-mimicking phantom: (**a**) Coronal projection results of a 10-mm target; (**b**) Coronal projection results of a 6-mm target; (**c**) 3D reconstruction results of the breast tissue-mimicking phantom.

For the KS105-HFR tissue-mimicking ultrasound phantom, two layers of data were selected for reflection tomography imaging, and the results are presented in Figure 11a,b. Quantitative analysis was conducted on the 10 mm and 6 mm cylindrical targets. Contrast was calculated based on the relative acoustic intensity difference between the cylindrical target regions and the background regions to determine the dimensions of the two cylindrical targets. The dimensional analysis results are shown in Figure 11c,d. Specifically, the reconstructed size of the 10 mm cylindrical target is 10.35 mm with a reconstruction error of 0.35 mm, while the reconstructed size of the 6 mm cylindrical target is 5.66 mm with a reconstruction error of 0.34 mm. The acquired breast-mimicking phantom data were processed for 3D reconstruction, and the results are displayed in Figure 12a shows the coronal projection of the cylindrical target with a diameter of 10 mm; Figure 12b shows the coronal projection of the cylindrical target with a diameter of 6 mm; and Figure 12c intuitively presents the two cylindrical targets of 10 mm and 6 mm in diameter.

### 4.3. Preliminary Clinical Feasibility Case Data Analysis

**Figure 13:**
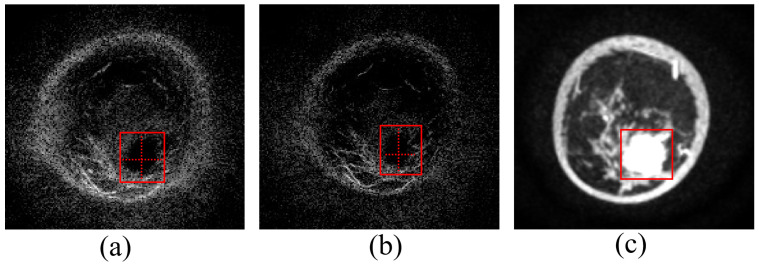
Lesion imaging: ultrasound reconstruction vs. MRI findings: (**a**) Ultrasound reconstruction image of the lesion at a sound speed of 1540 m/s; (**b**) Ultrasound reconstruction image of the lesion at a sound speed of 1513 m/s; (**c**) MRI findings of the lesion.

One layer of data from a volunteer with breast lesions was selected for ultrasound reflection tomography imaging, and the reconstruction results are presented in Figure 13. Specifically, Figure 13a shows the reconstruction result at a sound speed of 1540 m/s, Figure 13b displays the reconstruction result using the optimal estimated sound speed of 1513 m/s, and Figure 13c presents the MRI reconstruction result. The tumor regions under the two sound speed conditions were analyzed separately. The tumor region is the area selected by the red box in Figure 13. According to the Dice Similarity Coefficient (DSC) calculation, the reconstruction similarity at 1540 m/s was 0.69, while the reconstruction similarity using the optimal estimated sound speed of 1513 m/s was 0.87. These results indicate that the reconstruction similarity is significantly improved by the algorithm proposed in this study.

**Figure 14:**
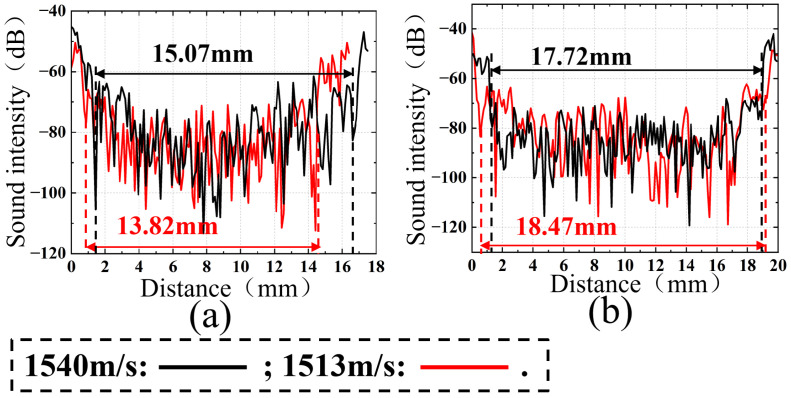
Quantitative analysis of lesion size at sound velocities of 1540 m/s and 1513 m/s: (**a**) Results of the quantification of the transverse dimensions of the lesion; (**b**) Results of the quantification of the axial dimensions of the lesion.

According to the results of the ultrasound examination report from the Chinese PLA General Hospital, the size of the mass is approximately 14 mm × 19 mm. Quantitative analysis was performed on the ultrasound reflection tomography image of this layer, and the results are presented in Figure 14. The black line represents the results obtained at a sound speed of 1540 m/s, while the red line represents those obtained using the optimal estimated sound speed of 1513 m/s. The lateral dimension analysis results are shown in Figure 14a: at 1540 m/s, the reconstructed dimension is 15.07 mm with an error of 1.07 mm; at the optimal estimated sound speed of 1513 m/s, the reconstructed dimension is 13.82 mm with an error of 0.18 mm. The longitudinal dimension analysis results are displayed in Figure 14b: at 1540 m/s, the reconstructed dimension is 17.72 mm with an error of 1.28 mm; at 1513 m/s, the reconstructed dimension is 18.47 mm with an error of 0.53 mm. These results demonstrate that the using of the optimal estimated sound speed effectively reduces the error of the reconstruction results.

**Figure 15:**
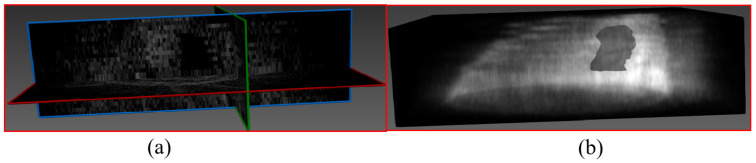
Visualization and 3D reconstruction of the breast lesion: (**a**) Coronal projection result of the lesion; (**b**) 3D reconstruction result of the lesion.

3D reconstruction was performed on the selected volunteer’s breast lesion to achieve visualization. The coronal projection of the breast lesion is presented in Figure 15a; the 3D reconstruction result is shown in Figure 15b, where partial morphological features of the breast lesion can be observed.

## 5. Conclusions

In this study, a high-resolution 3D ultrasound reflection imaging system for breast phantom and breast tissue analysis was successfully developed and validated through a preliminary clinical feasibility case study, demonstrating promising potential for the early detection of small breast lesions, as evidenced by a preliminary feasibility case study. In vitro experiments confirmed that using the optimal sound velocity estimation algorithm can improve the spatial resolution of the ultrasound imaging system from 1 mm to 0.5 mm; the target reconstruction error of 0.3 mm is reduced to 0.05 mm. Ultimately, the system achieves a spatial resolution of 0.5 mm and a target reconstruction error. Based on preliminary clinical feasibility studies of breast lesions, DSC calculations showed that the morphological similarity between the breast imaging results obtained using the optimal sound speed estimation algorithm and MRI results was improved from 0.69 to 0.87; the error in the lateral dimension of the lesion was reduced from 1.07 mm to 0.18 mm, and the error in the longitudinal dimension was reduced from 1.28 mm to 0.53 mm. Although these results preliminarily validate the system’s imaging performance, larger-scale clinical trials are still needed to confirm its stability and reliability.

This study has some limitations. First, although we used the Dice correlation coefficient to assess the overlap area between the MRI lesion region and the ultrasound-image-reconstructed lesion region, small lesions and slight boundary deviations can lead to a decrease in the Dice correlation coefficient, thus affecting the reliability of the ultrasound imaging system in clinical applications [34]. Therefore, future research should use deep learning-based image segmentation algorithms to capture fine boundary features to improve the reliability of the ultrasound imaging system in clinical applications. Second, sensitivity and specificity are more clinically significant diagnostic performance comparison indicators. However, as this study was a preliminary clinical validation, it only included one volunteer case for analysis, resulting in a sample size insufficient for statistical analysis of sensitivity and specificity. Future research will expand the clinical dataset and classify different types of lesions to supplement these diagnostic performance assessments.

In addition, ultrasonic waves undergo varying degrees of signal attenuation as they propagate through different soft tissues. Therefore, in future research, adaptive time gain compensation can be employed to dynamically adjust the compensation level based on the amplitude of real-time echo signals, thereby accommodating diverse tissue types and pathological lesions [35]. Current methods for sound speed correction also suffer from inherent limitations. Such methods compute a global sound speed across the imaging cross-section, which provides a balanced compromise for sound speed discrepancies among different tissues. Although these methods improve the overall image quality and resolution compared with the conventionally assumed sound speed of 1540 m/s, they fail to fully compensate for local phase aberrations induced by tissue heterogeneity. This issue is particularly pronounced at tissue boundaries with substantial sound speed disparities, severely compromising the reconstruction of fine structural details in these regions. To address the limitations of global sound speed estimation, future investigations should leverage deep learning to train models for predicting sound speed distributions. The focus should be placed on estimating the sound speed of individual tissue layers, thereby enabling the high-fidelity reconstruction of boundary structures. This imaging platform provides fundamental research for the detection of small lesions in the breast.

## Figures and Tables

**Table 1:** Imaging resolution body mode parameters.

Target	Target Diameter (mm)	Material Speed of Sound (m/s)
I	7	1500 ± 10
G	10	1550 ± 10
G	10	1550 ± 10
H	10	1600 ± 10
**Target Group**	**Target Interval (mm)**	**Target Diameter (mm)**
5	4, 3, 2, 1	0.3
5	3, 2, 1, 0.5	0.3

**Table 2:** Parameters of breast tissue-mimicking phantom.

Target	Target Diameter (mm)	Material Speed of Sound (m/s)
Background Organization	/	1540 ± 10
Columnar Mimic Tumor Foci	10	1540 ± 10
Pseudocalcified Lesion	5	1505 ± 10
Columnar Cystic Lesion	6	1540 ± 10
Irregular Cystic Lesions	/	1540 ± 10
Irregular Mimicry of Tumor Foci	/	1540 ± 10

**Table 3:** CR and gCNR values at different sound speeds.

Method	ROI4		ROI5		ROI9		ROI10	
	**CR**	**gCNR**	**CR**	**gCNR**	**CR**	**gCNR**	**CR**	**gCNR**
1540 m/s	1.36	0.91	1.08	0.88	1.44	0.86	1.37	0.87
1513 m/s	2.838	0.95	1.645	0.93	2.52	0.93	2.27	0.91

**Table 4:** Reconstructed target spacing and errors under different sound speeds.

Target Group	Calibration Distance (mm)	Target Distance of 1540 m/s (mm)	Inaccuracies	Target Distance of 1513 m/s (mm)	Inaccuracies
1–2	4	3.66	8.5%	3.84	4.0%
2–3	3	2.66	11.3%	3.08	2.6%
3–4	2	1.76	12.0%	1.92	4.0%
4–5	1	0.88	12.0%	1.07	7.0%
6–7	3	2.89	3.6%	3.06	2.0%
7–8	2	1.78	11.0%	1.97	1.5%
8–9	1	0.89	11.0%	1.07	7.0%
9–10	0.5	\	\	0.63	26.0%

**Table 5:** Linear measurement accuracy and precision: Note that the deviation value represents the measurement accuracy, calculated as the difference between the actual value and the measured value; the percentage deviation value is calculated as the percentage of the deviation value to the actual value. The target column identifies the numbered measurements in Figure 6a,b.

Sound of Speed	Target	Actual Value (mm)	Measured Values (mm ± SD)	%SD (Coefficient of Variation)	Bias (mm)	%Bias
1513 m/s	g	10.00	10.00 ± 0.32	9.4	0.32	3.2
1513 m/s	h	20.00	20.00 ± 0.44	2.9	0.44	2.2
1513 m/s	i	30.00	30.00 ± 0.52	2.7	0.52	1.7
1513 m/s	j	40.00	40.00 ± 0.66	1.9	0.66	1.6
1513 m/s	k	50.00	50.00 ± 0.78	1.9	0.78	1.6
average value				3.8	0.54	2.1
1540 m/s	g	10.00	10.00 ± 0.56	7.3	0.56	5.6
1540 m/s	h	20.00	20.00 ± 0.42	3.0	0.42	2.1
1540 m/s	i	30.00	30.00 ± 0.78	3.3	0.78	2.6
1540 m/s	j	40.00	40.00 ± 0.84	2.9	0.84	2.1
1540 m/s	k	50.00	50.00 ± 0.84	2.4	0.84	1.7
average value				3.8	0.69	2.8

**Table 6:** System Indicator Comparison.

Ultrasound Imaging System	Liu et al. [13]	Zhang et al. [14]	QT [31,32]	Softvue [33]	This Work
Sensor Components	Four 1 × 128 PMUT line arrays	1 × 256 ring transducer array	1 transmission transmitting array; 1 transmission receiving array; 3 reflection arrays	1 × 2048 ring transducer array	1 × 512 ring transducer array
Number of receive channels	64	256	2048	512	512
Number of transmit channels	64	1		512	1
Center Frequency	3.5 MHz	3 MHz	3.6 MHz	3 MHz	3 MHz
Array radius	90 mm	100 mm		110 mm	110 mm
Scanning Mode	Rotate every $2^{\circ }$ at equal intervals	Vertical rise at 1.5 mm equal intervals	moving vertically 2 mm		Vertical rise in 2 mm equal intervals
Duration of the scan		each frame of data lasts approximately 0.14 s	scan lasts 10 to 20 min	A complete breast scan takes approximately 2–4 min	Collecting data from one layer takes approximately 32 s
Spatial Resolution	10 mm	0.78 mm	Sub-millimeter		0.5 mm

## Data Availability

The data are available upon request from the authors.
